# Capturing Diabetes-Related Distress and Burden From the Perspective of Patients With Type 1 or Type 2 Diabetes: Protocol for an Explorative Mixed Methods Study

**DOI:** 10.2196/38477

**Published:** 2022-08-01

**Authors:** Sandra Zara, Johannes Kruse, Anna Maria Leveling, Jana Schmitz, Isabelle Hempler, Hanna Kampling

**Affiliations:** 1 Department of Psychosomatic Medicine and Psychotherapy Justus Liebig University Giessen Giessen Germany; 2 Department for Psychosomatic Medicine and Psychotherapy Medical Center of the Philipps University Marburg Marburg Germany; 3 Institute of General Practice / Family Medicine Medical Center – University of Freiburg Faculty of Medicine Freiburg Germany

**Keywords:** diabetes, diabetes-related distress, glycemic control, depression and anxiety, mixed methods design, mixed methods, protocol, stress, anxiety, depression, patient perspective, psychosocial, management, treatment, self-management

## Abstract

**Background:**

Diabetes is one of the most common diseases worldwide and is associated with increased morbidity, mortality, and reduced quality of life. Many patients experience high diabetes-related distress as well as depression and anxiety symptoms, which are associated with poor diabetes self-management. As disease management is a central component in diabetes treatment, poor management enhances the occurrence of micro- and macrovascular complications. This emphasizes the relevance of reducing diabetes-related distress and providing adequate treatment options addressing the individual psychosocial burden of patients with diabetes. Since patients’ perspectives diverge significantly from those of practitioners in terms of relevant treatment aspects, the patient perspective on, for example, barriers to and facilitators of diabetes treatment is crucial for adequate and effective treatment as well as improvements to self-management and therefore, needs to be further explored.

**Objective:**

This study aims to examine diabetes-related distress, the course of distress throughout diabetes management, as well as barriers and facilitating factors in dealing with diabetes from the individual perspective of patients with type 1 and type 2 diabetes.

**Methods:**

The study employs a mixed methods design combining qualitative and quantitative data. Semistructured interviews (N=40) will be conducted with patients with type 1 diabetes (n=20) and patients with type 2 diabetes (n=20). The primary outcomes comprise (1) diabetes-related distress, (2) the severity of distress, (3) the course of distress throughout diabetes management, (4) barriers, and (5) facilitating factors. Questionnaires will provide data on the following secondary outcomes: diabetes-related emotional distress (the Problem Areas in Diabetes scale), symptoms of depression and anxiety (Patient Health Questionnaire, German version), personality functioning (Operationalized Psychodynamic Diagnosis-Structure Questionnaire), mentalizing capacities (Mentalization Questionnaire), epistemic trust (Epistemic Trust, Mistrust and Credulity Questionnaire) and experiences of child maltreatment (Childhood Trauma Questionnaire), and the overall health status of the patient (routine medical data).

**Results:**

As of April 2022, the conceptualization phase of the study was finalized. Ethics approval was received in January 2022 from the local ethics committee of the Justus Liebig University Giessen – Faculty of Medicine (AZ 161/21).

**Conclusions:**

This study will provide insights into the individual perspective of patients with type 1 and type 2 diabetes regarding their experiences with diabetes management and what they perceive to be relevant, obstructive, or beneficial. The insights gained could help further tailor diabetes treatment to the individual needs of patients with diabetes and therefore optimize diabetes self-management.

**Trial Registration:**

German Clinical Trial Register DRKS00024999; https://tinyurl.com/2wb4xdh8

**International Registered Report Identifier (IRRID):**

PRR1-10.2196/38477

## Introduction

With a global prevalence of 476 million, diabetes is one of the most common diseases worldwide [1]. About 10% of the German population has diabetes [2], with projections indicating a further increase [3]. Diabetes is associated with increased morbidity, mortality, and reduced quality of life [4] and poses growing socioeconomic challenges for the health care system (eg, medication and hospitalization) [5]. Regarding treatment, adequate glycemic control (glycated hemoglobin [HbA_1c_] <7.5%) and a high degree of self-management are crucial. However, studies have shown that approximately 58.4% of patients with type 1 diabetes and 40.4% of patients with type 2 diabetes do not achieve their target glycemic control [6]. Additionally, as many as 42% of patients with type 1 diabetes and 24% to 36% of patients with type 2 diabetes report pronounced diabetes-related distress [7] (defined as the emotional aspect of the burden of living with a mainly self-managed chronic disease) [8]. High diabetes-related distress is associated with inadequate glycemic control (HbA_1c_>7.5%) as well as poor diabetes management [9,10], highlighting the relevance of reducing this burden for patients with diabetes.

Further, patients with inadequate glycemic control present elevated depression or anxiety symptoms [11]. Diabetes has been shown to increase the risk of depressive symptomatology [7], while, conversely, depressive symptomatology increases the risk of developing type 2 diabetes by 34% [12]. Patients with diabetes have about twice the risk of developing an anxiety disorder compared to patients without diabetes, with fears of acute complications, such as hypoglycemia or subsequent diseases and complications (microvascular diseases like retinopathies as well as macrovascular diseases) [13]. The presence of psychiatric comorbidity is linked to both morbidity and mortality and decreased quality of life [7], while health care costs and the risk of subsequent diseases are augmented [14]. Since psychological distress and psychiatric comorbidities not only require adequate treatment by themselves but also accelerate the development of diabetes-associated secondary diseases and worsen their course [15], they pose a significant treatment focus in patients with diabetes.

As strict monitoring and regulation of glycemic control are central in diabetes management, factors associated with poor glycemic control are important to consider. Evidence on the association between poor glycemic control and, for example, depression or anxiety symptoms is inconsistent. Some studies showed an association between depression and anxiety symptoms and poor glycemic control in adults with type 1 diabetes [16], while others found no association between depression and poor glycemic control [17,18]. This might be a conceptual issue, as the notion of diabetes-related distress better describes the psychosocial adjustment to diabetes than depression, comprising anger, guilt, frustration, denial, and loneliness [19]. Both constructs seem to overlap and correlate, yet are distinct [20,21], potentially explaining why pharmacological and psychological treatments for patients with depression and type 1 and type 2 diabetes yield inconsistent achievements regarding glycemic control [22,23]. Recently, a multidisciplinary, psychosomatic, psychodynamically oriented, short-term intervention improved glycemic control by focusing on individuals’ specific diabetes-related distress [24]. This emphasizes the need to align treatment with individual needs. Studies have shown that the focus of patients and practitioners diverges; patients mainly focus on the importance of diabetes in their daily life whereas practitioners almost exclusively orient toward measurable parameters [25]. This might hinder adequate diabetes management and affect the success of treatment options. Hence, to individualize interventions, further research on patients’ perspectives and their diabetes-related distress is needed.

The first aim of this study is to explore individual and specific issues in diabetes management in patients with poor glycemic control in order to better understand barriers and facilitators in their treatment. Based on qualitative data, we will derive patients’ perspectives on individual diabetes-related burdens, critical times in the course of their treatment, as well as barriers and support factors. The secondary outcomes will be measured with questionnaires addressing diabetes-related distress in the context of diabetes and treatment requirements, psychological aspects (depression and anxiety symptoms, personality functioning, mentalization capacities, epistemic trust, and experiences of child maltreatment), as well as physical health assessed through routine medical data (diabetes type; HbA_1c_; medication; weight and height; total cholesterol, low-density lipoprotein (LDL) and high-density lipoprotein (HDL) cholesterol, triglycerides; and previous illnesses).

## Methods

### Study Design

The study will be conducted using an explorative mixed methods design and will integrate qualitative as well as quantitative data. Patients between 18 and 69 years with diagnosed type 1 or type 2 diabetes, with an HbA_1c_ value >7.5%, a diabetes duration of minimum 2 years, a completed diabetes self-management program, sufficient German language skills, and cognitive abilities will be included in the study. Patients with type 3 diabetes or gestational diabetes, severe comorbid diseases (eg, dementia, major depressive disorder, psychosis, or addiction), a diabetic foot, and those who are bedridden or are care-dependent patients will be excluded from the investigation.

**Figure 1:**
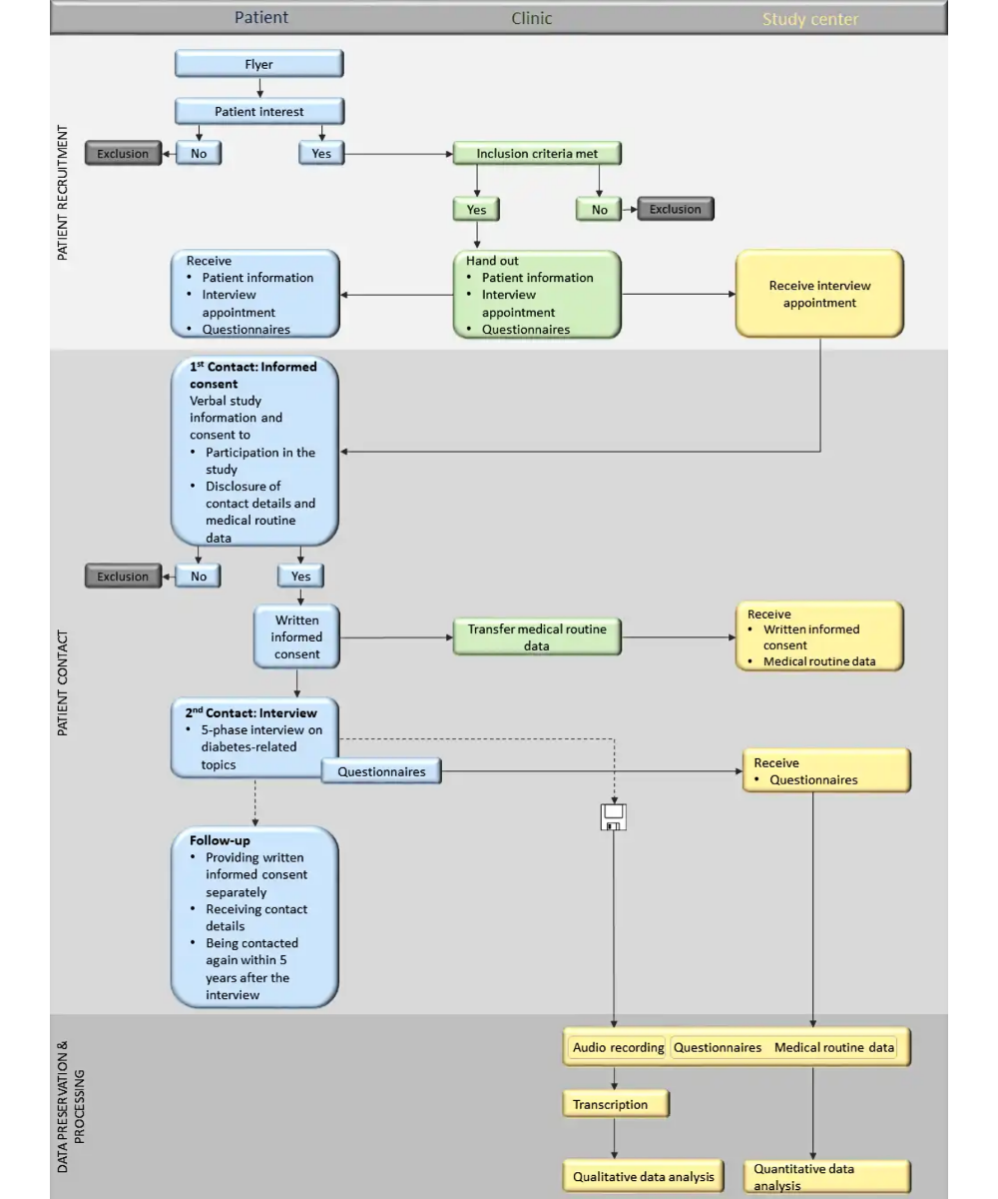
Schematic illustration of the study procedure.

Figure 1 illustrates the procedure of the study. Recruitment will take place in a clinic that specializes in diabetes. In step 1, the clinic will be equipped with all relevant documents (eg, information on the course of the study, data protection, form for assessing routine medical data, flyer and information for patients, and questionnaires). In step 2, patients will be asked by clinic personnel to participate in the study or made aware of the study by a flyer. In step 3, patients willing to participate in the study will be given comprehensive information, be informed verbally on the study purpose and procedure, and be assigned an interview appointment by the clinic personnel. In addition, self-report questionnaires will be handed out. In step 4, the researchers conducting the interviews will thoroughly discuss the patient information and the declaration of consent with the patient. After providing written informed consent, the interview will be conducted in the clinic. Due to the ongoing COVID-19 pandemic, the patient interviews will be conducted under strict compliance with the clinics’ hygiene regulations by researchers who are familiar with proper patient contact and the special regulations in effect under the pandemic.

### Sample Size

A total of 40 patient interviews (20 each for type 1 and type 2 diabetes) will be conducted. Among those, 4 interviews (2 each for type 1 and type 2 diabetes) are planned as a pretest to gain a deeper understanding of whether the developed guideline will work or if questions need to be reevaluated and modified.

### Assessment

#### Interview Guideline

Semistructured interviews will be conducted based on an interview guideline, with each section containing open questions, giving the participant the opportunity to speak openly and introduce new content.

##### Phase 1: Welcome

The participant will be greeted and—corresponding to the patient information—the interviewer will explain the interview as well as the protection of privacy, including pseudonymization of the data and how the transcript will be handled. After obtaining written informed consent and providing time for questions, the interviewer will start the audio recording and begin to collect sociodemographic information.

##### Phase 2 – Category I: Diagnosis and Course of Diabetes

This category aims to collect information on the patients’ experience with the initial diagnosis, difficult phases including symptoms of depression and anxiety, or diabetes-associated complications, as well as less difficult or good phases regarding diabetes. Sample questions include: “In your opinion, what went badly [when receiving the diagnosis]?” and “What memorable events were there in connection with diabetes and the treatment of diabetes (eg, complications and medication changes)?”

##### Phase 3 – Category II: Diabetes-Related Distress and Burden

In this category, individual diabetes-related distress and burden, including concerns and behavior changes, will be explored. Sample questions include: “What is it that worries you most about your diabetes?” and “What new experiences—positive and negative—have you had that you might not have had without diabetes?”

##### Phase 4 – Category III: Barriers and Facilitators

Questions of this category investigate barriers, for example, through doctors, the use of external support offers, as well as difficulties at work and in the social environment. Sample questions include: “What difficulties have arisen in the workplace?” and “Are there any tools (blood sugar diaries, food diaries, or apps) that make it easier for you to deal with diabetes?”

##### Phase 5: Final Phase

The participant will have the opportunity to tell the interviewer a specific recommendation that they believe to be useful for every patient with diabetes. After time for additional questions, the participant will be thanked for their time and participation.

#### Problem Areas in Diabetes Scale

The Problem Areas in Diabetes scale [26] includes 20 items to assess different areas of diabetes-related emotional distress. The response options range from 0 (“no problem”) to 4 (“major problem”). A sum score is computed and multiplied by 1.25, resulting in a total score between 0 and 100. Higher values represent more severe distress. A value >39 indicates severe emotional distress [19], pointing to significant depressive symptoms [26,27].

#### Patient Health Questionnaire

The Patient Health Questionnaire (German version; PHQ-D) measures depressive and anxiety syndromes, somatoform syndromes, eating disorders, alcohol abuse, psychosocial functioning, stressors, critical life events, menstruation, pregnancy, and childbirth, and shows good validity [28,29]. The depression module of the PHQ-D corresponds to the PHQ-9 [30]. By providing response options between 0 (“not at all”) and 3 (“almost every day”), it allows the calculation of a total score between 0 and 27, with severity classified as no depressive disorder, <5 points; mild depression, 5-9 points; moderate depression, 10-14 points; and severe depression, 15-27 points [30,31]. Anxiety syndromes are assessed with a panic module and a scale for other anxiety syndromes. The panic module consists of a general part on anxiety attacks (questions 3a-d) and a part with questions that refer specifically to the last severe anxiety attack (questions 4a-k). The possible answers are “yes” and “no.” A panic syndrome is expected when questions 3a to 3d are answered with “yes” and at least 4 of the questions 4a to 4k are answered with “yes.” Other anxiety syndromes (questions 5a-g) have the response options “not at all,” “some days,” and “more than half the days.” Another anxiety syndrome is assumed if question 5a is answered with “more than half of the days” and at least 3 of the questions 5b to 5g are answered with “more than half of the days” [32].

#### Childhood Trauma Questionnaire

Different forms of child maltreatment will be assessed with the Childhood Trauma Questionnaire (CTQ). Participants answer questions regarding sexual, emotional, and physical abuse as well as emotional and physical neglect on a scale with response options ranging from 1 (“not at all”) to 5 (“very often”). Each subscale consists of 5 items, resulting in sum scores from 5 to 25. Severity is classified as none to minimal, low to moderate, moderate to severe, and severe to extreme [33]. The German version of the CTQ [34] shows good internal consistency, except for physical neglect (sexual abuse: α=.89; physical abuse: α=.80; emotional abuse: α=.87; physical neglect: α=.55; and emotional neglect: α=.83).

#### Operationalized Psychodynamic Diagnosis-Structure Questionnaire

The Operationalized Psychodynamic Diagnosis-Structure Questionnaire (OPD-SQS) is a self-report questionnaire to screen for participants with deficits in personality functioning. It comprises 3 subscales (ie, self-perception, interpersonal contact, and relationship model) with 4 items in each scale. Response options range from 0 (“does not apply at all”) to 4 (“fully applies”), resulting in a sum score from 0 to 48, with higher scores indicating impairments in personality functioning. The OPD-SQS showed good internal consistency (α=.88) [35].

#### Mentalization Questionnaire

The Mentalization Questionnaire [36] is a self-report instrument to assess mentalization capacities from the patient’s perspective. It consists of 15 items with response options ranging from 1 (“no agreement at all”) to 5 (“total agreement”). Analyses yielded 4 subscales with acceptable reliability and sufficient validity: (1) refusing self-reflection, (2) emotional awareness, (3) psychic equivalence mode, and (4) regulation of affect. The sum score ranges from 15 to 75, with higher scores indicating lower mentalization capacities.

#### Epistemic Trust

The Epistemic Trust, Mistrust and Credulity Questionnaire by Campbell and colleagues [37] was developed as a self-report questionnaire to assess epistemic trust, distrust, and gullibility. It comprises 15 items with response options ranging from 1 (“strongly disagree”) to 7 (“strongly agree”) [38], resulting in a score from 15 to 105. High epistemic trust, mistrust, and credulity are indicated by either strong agreement or strong disagreement with the statement.

#### Routine Medical Data

Routine medical data collected at doctor’s appointments comprise diabetes type (type 1 or 2), HbA_1c_ level, and current medication. Weight and height to calculate BMI; total cholesterol, LDL cholesterol, HDL cholesterol, and triglycerides to assess lipid metabolism; and information on preexisting conditions (eg, coagulation disorders, cardiovascular disease, neuropathy, retinopathy, peripheral arterial occlusive disease) to assess the patients’ health status will be collected.

### Outcome Parameters

Primary outcomes: qualitative data from semistructured interviews will comprise (1) diabetes-related distress; (2) severity of diabetes-related distress; (3) general distress at the time of diagnosis notification, medication change, if applicable, and special events regarding the patients’ social environment; (4) barriers; and (5) facilitating factors. Secondary outcomes include quantitative data that will comprise self-report questionnaire data to assess (1) diabetes-related emotional distress and (2) psychological aspects (depression and anxiety symptoms, personality functioning, mentalization capacities, epistemic trust, and child maltreatment), as well as (3) routine medical data (diabetes type, HbA_1c_, medication, weight, height, total cholesterol, LDL and HDL cholesterol, triglycerides, and preexisting conditions).

### Data Analysis

#### Qualitative Data Analysis

The audio recordings of the semistructured interviews will be transcribed. During the transcription process, all personal data (including that of third parties) will be made unrecognizable. The pseudonymized transcript will then be analyzed by means of content structuring qualitative content analysis [39] using MAXQDA (VERBI GmbH). The material will be systematically described with regard to individual categories that are determined in connection with the research question and differentiated during the analysis. Special emphasis will be placed on text comprehension and text interpretation [39]. The evaluation of the transcript will be divided into the following steps:

1. Development of main topics for the semistructured interview guideline
2. Initiating text work on the material
3. Inductive determination of main categories
4. First coding process
5. Compilation of main categories
6. Inductive determination of subcategories on the material
7. Second coding process
8. Simple and complex analyses and visualizations

#### Quantitative Data Analysis

The descriptive exploratory statistical analyses of the quantitative data (questionnaire data and routine medical data) will be performed using SPSS Statistics (IBM Corp).

### Ethics Approval

Ethical approval was obtained from the Ethics Committee of Justus Liebig University Giessen – Faculty of Medicine (AZ 161/21). The study is registered in the German Clinical Trial Register (DRKS00024999). All personal data of the participants are subject to medical confidentiality, the German general data protection regulation (Datenschutz-Grundverordnung), and state and federal data protection acts (Landesdatenschutzgesetz and Bundesdatenschutzgesetz). To maintain anonymity, the data will be pseudonymized and the corresponding codes will be kept by the principal investigator. The data will be stored for up to 5 years after final publication.

## Results

As of April 2022, the conceptualization phase of the study conduct has been finalized.

## Discussion

### Expected Findings

This study aims to gain insights into the individual perspective of patients with type 1 and type 2 diabetes on their experiences with their diabetes diagnosis, diabetes-related distress and burdens, psychosocial aspects, and barriers and facilitators, as well as what they perceive to be particularly relevant, obstructive, or beneficial regarding these subject areas. With the applied mixed methods design we expect to comprehensively explore individual diabetes-related burdens and facilitating factors adding to the numerous well-known challenges of patients with diabetes and, hence, inform diabetes treatment as well as focus on important psychosocial aspects for successful treatment. The results of our study will lay the groundwork for a new questionnaire to systematically assess individual diabetes-related distress, burdens, and facilitators that are useful for diabetologists by informing treatment planning as well as for future research in this field by enabling the systematic assessment of individual challenges and problem areas.

### Limitations

Regarding the proposed methodology of this study, a number of possible limitations must be acknowledged. First, due to recruitment taking place in a diabetes clinic, our study population might face particular challenges compared to patients with diabetes who receive outpatient treatment, potentially limiting the generalizability of our findings. Further, we omitted patients with gestational diabetes and other diabetes types. Mixed methods research generally faces the conceptual challenge of how methods should be selected for a given research question, what the mixing of approaches refers to, and, eventually, how a mixed methods methodology should be structured [40]. Further, an immanent part of qualitative research is the possibility of receiving socially desired answers, especially when it comes to sensitive issues. With regard to the time frame of the patient interview (approximately 60 minutes), addressing all aspects that might be of interest will not be possible. For example, suicidal ideation will not be specifically addressed. To account for this, we will end each interview subject with open questions, giving the patient the opportunity to bring subjects to our attention.

### Practical Implications

Based on our results, we aim to expand the knowledge about common diabetes-associated challenges and burdens as well as resources by exploring individual and potentially less evident problems from the patient perspective. The findings will be translated into a questionnaire allowing both practitioners and researchers to individually, efficiently, and systematically assess diabetes-related burden and subsequently inform treatment planning regarding the psychological as well as diabetological aspects to improve diabetes treatment.

## Abbreviations

CTQ: Childhood Trauma Questionnaire
HbA_1c_: glycated hemoglobin
HDL: high-density lipoprotein
LDL: low-density lipoprotein
OPD-SQS: Operationalized Psychodynamic Diagnosis-Structure Questionnaire
PHQ-9: Patient Health Questionnaire–9
PHQ-D: Patient Health Questionnaire, German version

## Data Availability

Data sharing is not applicable to this paper as no data sets were generated or analyzed during the study.
